# Exploring Volatile Profiles and De-Flavoring Strategies for Enhanced Acceptance of Lentil-Based Foods: A Review

**DOI:** 10.3390/foods13162608

**Published:** 2024-08-20

**Authors:** Francesca Vurro, Davide De Angelis, Giacomo Squeo, Francesco Caponio, Carmine Summo, Antonella Pasqualone

**Affiliations:** Department of Soil, Plant and Food Science (DISSPA), University of Bari ‘Aldo Moro’, Via Amendola, 165/a, 70126 Bari, Italy; francesca.vurro@uniba.it (F.V.); davide.deangelis@uniba.it (D.D.A.); giacomo.squeo@uniba.it (G.S.); carmine.summo@uniba.it (C.S.)

**Keywords:** legume, *Lens culinaris* Medik., volatile compounds, off-flavor, food processing, aroma, odor, plant breeding

## Abstract

Lentils are marketed as dry seeds, fresh sprouts, flours, protein isolates, and concentrates used as ingredients in many traditional and innovative food products, including dairy and meat analogs. Appreciated for their nutritional and health benefits, lentil ingredients and food products may be affected by off-flavor notes described as “beany”, “green”, and “grassy”, which can limit consumer acceptance. This narrative review delves into the volatile profiles of lentil ingredients and possible de-flavoring strategies, focusing on their effectiveness. Assuming that appropriate storage and processing are conducted, so as to prevent or limit undesired oxidative phenomena, several treatments are available: thermal (pre-cooking, roasting, and drying), non-thermal (high-pressure processing, alcohol washing, pH variation, and addition of adsorbents), and biotechnological (germination and fermentation), all of which are able to reduce the beany flavor. It appears that lentil is less studied than other legumes and more research should be conducted. Innovative technologies with great potential, such as high-pressure processing or the use of adsorbents, have been not been explored in detail or are still totally unexplored for lentil. In parallel, the development of lentil varieties with a low LOX and lipid content, as is currently in progress for soybean and pea, would significantly reduce off-flavor notes.

## 1. Introduction

Since ancient times, legumes, also known as “the poor man’s meat”, have been the main source of protein in the Mediterranean diet. Among them, chickpeas (*Cicer arietinum* L.), fava beans (*Vicia faba* L.), lentils (*Lens culinaris* Medik.), peas (*Pisum sativum* L.), and lupins (*Lupin* spp.) are widely cultivated and consumed [1,2,3]. Due to the promotion of new dietary lifestyles (e.g., flexitarian, vegetarian, and vegan diets) and the increasing awareness of reducing meat consumption for environmental and health reasons, the demand for legumes and legume-based products is expected to grow significantly, with a compound annual growth rate (CAGR) of 5.3% over the period of 2023–2032 [4].

A comprehensive analysis of global lentil production from 1961 to 2022 reveals a steady upward trend, surging up to eightfold and reaching 6.66 million tons in 2022, according to FAOSTAT (2023) [5]. Lentils have gained prominence in the marketplace, being recognized by consumers for their healthy nutritional profile and versatility of use, making them an economical meal solution that is widely consumed around the world [6]. In fact, lentils can be consumed as dried seeds, characterized by the shortest cooking time among legumes, or as fresh sprouts [7]. Dried seeds are boiled to prepare soups, curries, or salads, such as *masoor* in Asian countries, *mujaddara* in Arab countries, *cotechino con lenticchie* in Italy, and *koshari* and *hlalem* in the Mediterranean region. They can be used as a base for vegetarian balls, *hummus*, and pancakes [8,9]. In addition, lentils can be made into flour, which can be used to prepare popular dishes such as *papad* (or *papadam*), a South Asian fried cracker [8,10]. Lentil flour and lentil protein can be used as ingredients in many other food products, including baked goods [11,12,13], pasta [14], extruded snacks [15,16], protein drinks [17], plant-based meat analogs [18], and dairy alternatives [19,20].

Compared to cereals, lentils are higher in protein and lower in carbohydrates, with variations in composition due to genetic differences, environmental factors, and agronomic practices [6]. Their carbohydrate content ranges from 43% to 75% and has a limited impact on the glycemic index due to the presence of slowly digestible starch and fibers (5–27%). Their protein content ranges from 16% to 31%, with the amino acid profile dominated by glutamic acid, aspartic acid, arginine, leucine, and lysine. Conversely, cystine, tryptophan, and methionine are present in lower proportions [6]. Therefore, the common practice of combining lentils with cereal-based foods (rice, bread, or pasta) balances the amino acid profile and is nutritionally optimal. In addition, lentils are low in fat (1.0–3.5%) and high in minerals (2–6.5%) [6,21], contributing to the dietary intake of iron (Fe), zinc (Zn), and selenium (Se) [10]. Lentil cotyledons are also rich in bioactive compounds such as flavonoids, carotenoids, and tocopherols, recognized for playing a role in preventing oxidative stress, cellular damage, and several pathological conditions [6].

However, some negative implications associated with lentil consumption should also be considered. Lentils contain antinutritional compounds (e.g., trypsin inhibitors, phytates, raffinose-family oligosaccharides, and tannins) that reduce the digestibility of nutrients or cause gastrointestinal discomfort and flatulence [6]. These antinutritional compounds may be reduced by cooking or fermentation [22]. In addition, lentils have high lipoxygenase activity (LOX, E.C. 1.13.11.12), which is responsible for the oxidation of polyunsaturated fatty acids [23,24,25]. As a result, off-flavors may arise, adversely affecting the sensory quality of lentil derivatives [7].

Perceptible flavor notes in lentils can be intrinsic, such as “beany” (reminiscent of all boiled legume seeds), “green” (reminiscent of green pea), and “grassy” (reminiscent of cut grass) notes, or they can be developed during processing and storage, such as the typical rancid note of aged oil [7] caused by lipid oxidation [26]. Off-flavors in legumes and legume-based foods are a well-documented phenomenon [27,28,29]. The beany off-flavor is particularly noted by consumers as an unfamiliar flavor that limits the acceptability of foods containing legume flours or legume proteins. Reported as early as the late 1970s [30], the problem of the beany off-flavor is still a hot topic today [31] because of the global shift towards legume-based foods. Han et al. [32] developed a gluten-free cracker snack using green and red lentils mixed with other flours and proteins. A strong beany flavor was reported by many consumers. Ryland et al. [33] formulated a snack bar containing micronized flaked lentils, but the lentil flavor, among other attributes, had a great influence on consumer acceptability.

Strategies for reducing the off-flavors of pea and soybean have been studied in order to provide the food industry with sensory-neutral ingredients from these legumes [34,35,36,37]. However, there is a significant gap in the scientific literature regarding lentil de-flavoring. Despite the popularity of this legume, studies on its volatile profile and on techniques for reducing off-flavors remain fragmented. The reviews published on lentils so far have focused only on their nutritional, techno-functional, and health properties [38,39,40,41,42]. In this context, this review delves into the origins of the volatile compounds of lentils, examining the current state of research. De-flavoring strategies are described, focusing on their effectiveness and potential applicability in the food industry context.

## 2. Methodology of the Review and State of the Art

A traditional narrative review approach was adopted to critically summarize studies on the volatile compounds responsible for the off-flavors in lentils and the main strategies for achieving their reduction. A literature search and data collection were carried out by accessing scientific databases (Scopus, Web of Science, PubMed, and Google Scholar) and considering all articles that contained the keyword combinations “lentil” and “flavor”/“off-flavor”/“volatile compounds” in the article title, abstract, or keywords, published from January 2016 to June 2024. Supplementary searches were then carried out on specific themes that emerged in the literature, such as innovative de-flavoring techniques proposed for other pulses that could also be useful for lentils.

**Figure 1:**
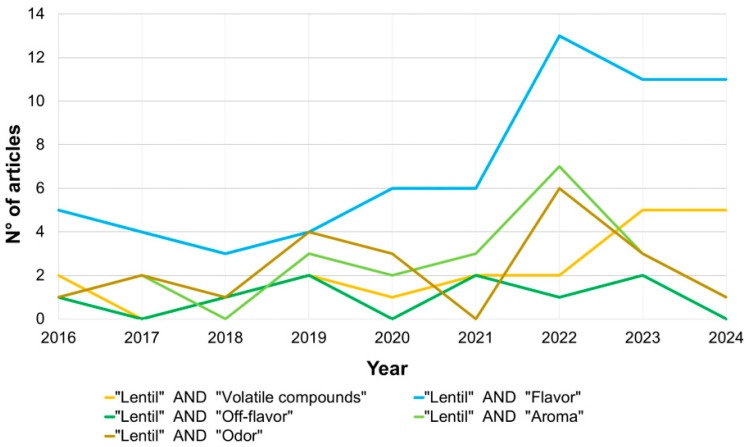
Number of published articles in the years 2016–2024 showing “lentil” and “flavor”/“off-flavor”/“volatile compounds/“aroma”/“odor” in the field “article, abstract, and keywords” (Own elaboration on data retrieved from the Scopus database).

Figure 1 shows the trend of articles published between 2016, declared the “International Year of Pulses” by the Food and Agriculture Organization of the United Nations (FAO), and June 2024. Over recent years, there has been a significant increase in the number of articles regarding “lentil” and “volatile compounds”. A particularly high number of articles with the words “lentil” and “flavor” can be observed in 2022. Instead, the words “odor” and “aroma”, which refer to orthonasal olfaction (while flavor is retronasal olfaction), were less present in the scientific literature.

## 3. Flavor/Aroma Notes Typically Present in Lentils and Their Origins

Table 1 lists the principal flavor/aroma notes and their associated volatile compounds identified in lentil flours, protein isolates, concentrates, and derived foods. All these flavor/aroma notes are undesirable and limit acceptability by consumers, who generally prefer more sensory-neutral lentil-derived products [33].

The main off-flavor of lentils, named “beany” (or named “legumy” in early papers), is an unpleasant note described as a combination of multiple attributes, including musty, earthy, dusty, sour, green, sweaty, nutty, and brown [44]. This sensory note, inherent in fresh, raw, legumes, is due to plant metabolism and, later, to enzymatic degradation during harvesting, storage, and processing. According to Troszyńska et al. (2011) [7], the reference for this sensory note is a suspension of lentil seeds in hot water 1:3 (*w*/*v*). The beany flavor results from the combination of aldehydes, alcohols, ketones, and furans naturally present in the seeds or formed through chemical reactions occurring during processing. The presence of a single molecule alone does not generally explain this unpleasant beany flavor. The list of compounds considered to be responsible for this broad and complex flavor note may vary among studies, also because every volatile compound can change its sensory effect depending on its concentration. Vara-Ubol et al. (2004) [44] observed that 3-methyl-1-butanol, 1-pentanol, (*E*,*E*)-2,4-heptadienal, acetophenone, and 1-octen-3-one are responsible for a beany note when their concentration is ≤10 ppm, changing to “sweaty” (for 3-methyl-1-butanol and 1-pentanol), “barnyard, manure” (for 1-pentanol), “rancid, fishy” (for (*E*,*E*)-2,4-heptadienal), or “musty/earthy, floral, sweet” (for acetophenone and 1-octen-3-one) at higher concentrations, while 1-octen-3-ol provides a beany flavor at concentrations between 100 and 1000 ppm. Xu et al. (2019) [45] defined hexanal, (*E*,*E*)-2,4-nonadienal, (*E*,*E*)-2,4-decadienal, 3-methyl-1-butanol, 1-hexanol, and 2-pentyl-furan as beany flavor markers. Further studies by other authors [35] pointed out that some compounds, such as hexanal, (*E*)-2-octenal, (*E*)-2-nonenal, (*E*,*E*)-2,4-nonadienal, (*E*)-2-hexenal, (*E*,*E*)-2,4-decadienal, and pentanal, are responsible for off-flavors (namely “rancid”), but do not have a beany flavor, *per se*, and should be considered as “non beany”. However, these compounds are able to enhance the intensity of the beany note. The situation is further complicated by the fact that the same authors observed that combinations of these “non-beany” volatiles may ultimately result in a beany flavor [35].

The aforementioned volatiles, including aldehydes, alcohols, ketones, and furans, linked to the “beany” flavor/aroma note (or, according to Trindler et al. [35], to a “non-beany” off-flavor, that is, “rancid”) result from the degradation and oxidation of lipids. The main event they arise from is the physical breakdown of cell tissues, causing the release and activation of lipase and LOX. Lipase releases free fatty acids from triglycerides, then LOX prompts the oxidation of linoleic and linolenic acids with the production of C6 and C9 aldehydes and alcohols [23,46], with the latter coming mainly from aldehyde reduction by the activity of alcohol dehydrogenase (ADH) [28]. The enzymatic activity of LOX also alters pigments (carotenoids and chlorophyll), resulting in off-flavors and discoloration [23]. Alternatively, the autooxidation of linoleic and linolenic acids may occur [23,46].

Methyl ketones (such as 2-butanone, 2-pentanone, and 2-hexanone) are generated through the decarboxylation of 3-oxo free fatty acids or via the aldol condensation of an aldehyde and an aldol intermediate [29]. 2-Pentyl furan, the most common representative lipid-derived furan, arises from the autoxidation and cyclization of linoleic acid or from the thermal interaction of (*E*,*E*)-2,4-decadienal with cysteine [28,29].

A key compound formed by linoleic acid is hexanal, an aldehyde considered to be a marker of oxidation in food matrices [26,47]. Hexanal is associated with notes described as “green” (sensory reference = green seeds of peas [7]), “grassy” (sensory reference = 1% (*Z*)-3-hexen-1-ol, [7]), and “rancid”, together with other aldehydes such as heptanal and octanal [26,48]. Green and grassy notes are typical of lentil sprouts [7].

Other studies, despite not being carried out specifically on lentils, also assessed that some pyrazine derivatives, which can be the products of protein and amino acid degradation, are responsible for “beany” and/or “green” flavors [35,37,49], such as 3-isobutyl-2-methoxypyrazine and 2-isopropyl-3-methoxypyrazine. In contrast, pyrazines such as 2,5-dimethy-lpyrazine and 3-ethyl-2,5-dimethylpyrazine have a pleasant “nutty” or “roasted” flavor [27,34,50].

The “brothy” flavor (sensory reference: one stock cube in 2 L of boiling water [51]) is another note identified in the lentil flavor profile, related to the presence of methional derived from methionine by Strecker degradation, which imparts an off-flavor reminiscent of cooked potatoes and cabbage [52]. Dimethyl sulfide and dimethyl trisulfide recall metallic, cabbage, egg, and onion off-flavors, are highly disliked by consumers, and can be defined as a “sulfur” note [26].

Over the years, researchers have often reported the negative sensory effects of fortifying food products with legumes, suggesting the need to find viable solutions to overcome these problems. Unsurprisingly, both traditional and innovative lentil-based recipes often include herbs and spices such as turmeric, ginger, oregano, bay leaf, and rosemary, which are rich in palatable essential oils, to mask the beany off-flavor [53,54,55]. However, with a thorough understanding of the effects of basic processing steps and storage on the volatile profile of lentil flours, actions should be taken to select the appropriate conditions for seed treatment and storage, flour production, and any other applied processes, especially to avoid or mitigate off-flavors [35].

## 4. Effect of Storage and Basic Processing on the Volatile Profile

### 4.1. Storage

Storage may greatly affect the volatile profile of lentils. Factors such as temperature, exposure to oxygen and light, and moisture content influence the kinetics of chemical reactions. Low temperatures and/or a low moisture content are essential for the storage of any bulk grain, preventing mold and oxidation, and mitigating the formation of off-flavors. Red lentils stored at 10 °C and 20 °C for 16 weeks showed no significant increase in free fatty acids (ready to be oxidized), even having a moisture content of 17.5%. If the moisture content decreased to 10%, the lentils showed a limited increase in free fatty acids during storage at 40 °C for up to 10 weeks, while samples with a 12.5% moisture content tripled their free fatty acids in the same storage time [56]. This study did not evaluate volatile compounds, but in another legume, peas, a moisture content of less than 10% prevented the onset of unpleasant odors for up to one year of storage at 30 °C [35]. For red lentil, buyers and processors prefer a seed moisture content of 13% to facilitate dehulling and splitting, but for “safe” storage (absence of discoloration, off-flavors, mold, and insect proliferation) for up to 28 weeks, the temperature must not exceed 20 °C or, to reach 50 weeks, 15 °C [57].

Low temperatures can further extend storage time. Lentils with a moisture content of 13% can be stored for up to 175 weeks at 5 °C [58], but no details were given by the authors on the effect of such prolonged storage on flavor. Raw peas stored at 4 °C for 12 months showed lower contents of aldehydes and sulfur compounds than those stored at 22 °C due to reduced LOX activity at low temperature [59]. More specific studies would be needed on lentil to assess the effect of storage conditions on the evolution of the volatile compounds responsible for the onset of off-flavor.

### 4.2. Dehulling and Milling

Lentils are first subjected to conditioning, which is a moistening treatment to soften their hulls and facilitate their subsequent removal. Dehulling and milling are then carried out to separate the outer layers of the seed, known as the hull, from the inner edible portion (cotyledon), subsequently reducing the latter to smaller particles or flour [60].

This series of mechanical operations, leading to certain temperature increases due to friction occurring during and to relevant exposure to oxygen, can affect the flavor profile of lentils [35]. The removal of the hulls of brown and green lentils resulted in variations in their volatile profiles, with a reduction in non-terpene volatiles and the formation of phenylpropanoids and apocarotenoids [43]. The dehulling of lentils has been reported to improve their flavor [58] and reduce the content of tannins, mainly responsible for a bitter sensory note [6]. Therefore, today, lentils are commercialized mainly in dehulled form.

Oxidation compounds such as nonanal and 2-methyl-1-hexanol increase with milling [35]. Rajhi et al. [43] confirmed that milling enhances exposure to oxygen and increases the intensity of the beany flavor of lentils. Tissue damage and enzyme release, causing volatile production by enzymatic mechanisms, are unavoidable during processing, but can be minimized. For instance, peas milled to a 500 µm average particle size showed lower levels of volatile compounds than those milled to 250 µm [61]. Similar studies should be conducted on lentils to define the optimal conditions to reduce the off-flavors related to milling.

## 5. De-Flavoring Strategies

**Figure 2:**
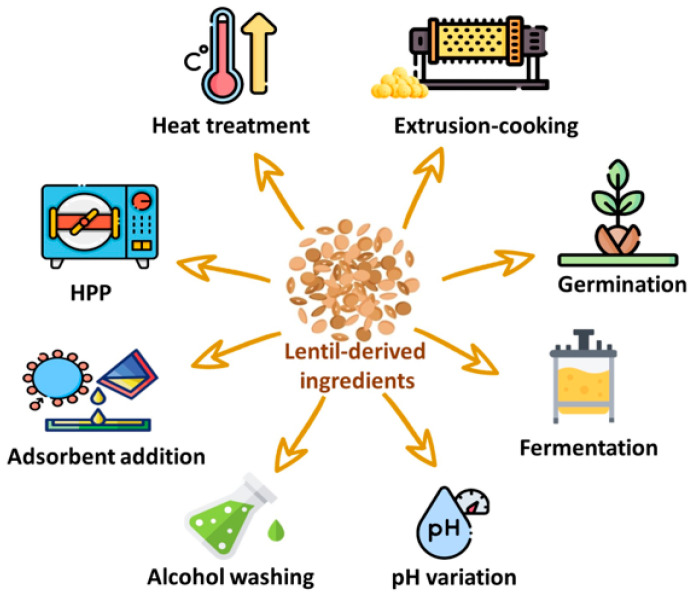
De-flavoring strategies applicable to lentil seeds, flours, or protein isolates and concentrates. HPP = high-pressure processing.

The treatments that can be undertaken to reduce the off-flavors in lentil flours and derived food ingredients and products are schematized in Figure 2, while their main mechanisms of action, advantages, and disadvantages are summarized in Table 2.

Depending on the type of treatment, there may be changes in the volatile profile due to physical–chemical modifications of components (e.g., protein and enzyme—including LOX—denaturation); chemical interactions between molecules (e.g., Maillard reaction leading to new flavors); and the removal or adsorption of the compounds responsible for undesirable flavors. Some treatments may lead to a decrease in LOX activity, resulting in a reduction in oxidation compounds (e.g., heat treatments, high pressures, use of solvents, or pH changes), while others may lead to the formation of new chemical compounds that enrich the volatile profile and may mask the perception of off-flavors (e.g., fermentation, germination, or extrusion cooking).

However, economic and environmental issues must be considered in the view of practical applications, because some treatments (such as HHP and extrusion cooking) require significant investments in equipment, energy, and/or water, while others are easily replicable without relevant additional costs. Finally, some treatments, such as alcohol washing, pH variations, and adsorbent addition, require special attention to avoid the possible presence of chemical residues in food, by adopting purification steps and exclusively using food-grade chemicals.

### 5.1. Heat Treatments

#### 5.1.1. Pre-Cooking, Roasting, and Drying

The most common heat treatments for lentils are pre-cooking (boiling or steaming), roasting, and drying. These treatments can involve the application of microwave and radio frequencies to achieve the desired outcomes, such as specific changes in physical–chemical properties, making food more digestible and palatable. The heat treatment of lentil seeds and flour denatures LOX and possibly peroxidase, reducing their activity. However, the thermal process may promote the autoxidation of unsaturated free fatty acids [62]. Another effect of thermal treatments, such as roasting, is that they produce new volatile compounds derived from the Maillard reaction, such as furans and pyrazines, that can mask the beany flavor.

Shariati-Ievari et al. [75] investigated the effect of infrared heat treatment on LOX activity and, consequently, on the volatile compounds of green lentils while being micronized. A significant reduction in LOX activity was observed after treatment at 130 °C, and a further decrease at 150 °C, compared to untreated flour, with a reduction in the hexanal and 2-hexenal levels. Burgers prepared with heat-treated lentil flour had a good overall acceptability and flavor, while those prepared with untreated flour were characterized by a beany off-flavor. Also, blanching and steaming are effective in deactivating LOX and decreasing the off-flavors in pulses, such as peas and beans [76], although there have been no studies carried out on lentil.

Different heat treatments, such as roasting, pre-cooking, and drying, have different effects on the flavor profile: roasting increases and complexifies the volatile profile, while pre-cooking and drying decrease the flavor intensity. In detail, the roasting of lentil seeds for 20 min at about 100 °C reduced the total alcohols, did not change the total aldehydes, and increased the pyrazines compared to raw lentils, with an overall increase in the total volatile compounds. In contrast, lentil pre-cooking (by boiling for 20 min) markedly reduced the total volatiles compared to raw lentils by lowering the level of total alcohols, without affecting the total aldehydes [46].

Drying is the final stage in the preparation of pre-cooked lentil flours or wet-fractionated lentil proteins, and is required after the fermentation of lentils and before their grinding when preparing fermented flours [77]. For preparing pre-cooked lentil flours, cooked seeds must be mashed in a blender with water and finally spray-dried. Drying (by setting the inlet and outlet temperature of the spray-drier at 130 and 80 °C, respectively) was found to slightly increase the total volatiles compared to pre-cooked seeds, but these still remained below the concentration found in raw lentils. Alcohols were reduced by drying, but aldehydes (especially hexanal) strongly increased compared to the raw lentils [46].

Drying also reduced the total volatiles of fermented lentils. Fermentation tends to enrich the volatile profile (see Section 5.7), but the total sum of volatiles decreased after hot-air drying. Alcohols and aldehydes (particularly 1-octene-3-ol, hexanal, benzaldehyde, and 3-methoxybenzaldehyde) were the chemical compounds that varied the most. These compounds decreased progressively with an increasing temperature (from 50 to 70 °C) [77]. The loss of some volatile compounds at higher temperatures is due to thermal degradation and volatilization [78]. In contrast, pyrazines were generated by the Strecker degradation and Maillard reactions, resulting in pleasant caramel-like, burnt, and cooked notes [79]. As a result, combined with fermentation, hot-air drying improved the flavor of lentils.

Innovative techniques should be also considered, such as microwave and radio frequency heating, which were found to effectively inhibit the LOX activity of soybean in a very short time, de-flavoring the soybean protein [80,81]. Microwaves were also used to set up a dehydration system working in vacuum, which was able to remove flavors from plant-based non-dairy beverages [82]. These techniques have not been applied to lentil ingredients so far.

#### 5.1.2. Extrusion Cooking

Extrusion cooking is a versatile thermo-mechanical process widely used in the lentil value chain to produce snacks, textured proteins, and meat analogs [65,83,84]. The deactivation of LOX occurs at temperatures above 100 °C, like those adopted during extrusion cooking, but requires sufficient time. During extrusion cooking, which is usually a relatively fast process (with the residence time in the extruder cooker being between 2 and 10 min) [64], residual LOX activity and high exposure to oxygen may significantly affect the lipid fraction generating the compounds responsible for off-flavors [23]. The chemical composition of the starting flour also has an influence. In fact, during the production of legume-based extruded snacks, some off-flavors, such as “fatty/cardboard-like” and “fatty/deep-fried”, were rated lower in lentil snacks than in blue lupin snacks, opposite to a “popcorn-like” flavor, which was scored higher in the lentil snacks [83].

Temperature and screw speed, the two main parameters set during the process, were found to affect the volatile compounds. During the production of lentil-based high-moisture meat analogs, nonanal and benzaldehyde increased by raising the temperature to 150 °C, but decreased at values of >160 °C. Accordingly, the odor concentration decreased as the temperature increased [65]. Similar results were found when studying the effect of the extrusion temperature on the flavor of texturized soybean proteins. The authors hypothesized that increased flash evaporation at the exit of the die, occurring at higher temperatures, caused a loss of volatile compounds [85]. In detail, the levels of 2-pentyl-furan, 2-nonanone, 1-octen-3-ol, nonanal, hexanal, benzaldehyde, and 2-heptanone decreased at temperatures of >155 °C [85].

Screw speed showed a similar effect to temperature. The total flavor compounds of lentil-based meat analogs were more concentrated when the screw speed was raised from 800 to 1000 rpm, but decreased at values of >1000 rpm [65]. Therefore, at certain settings of temperature and screw speed, extrusion cooking can contribute to the reduction in flavors.

### 5.2. High-Pressure Processing (HPP)

High-pressure processing (HPP) is a non-thermal technology that relies on applying a pressure between 300 and 600 MPa to food for a few minutes. The treatment is performed in a vessel where the product is immersed in water, responsible for the instantaneous transmission of pressure throughout the product [66]. HPP offers the possibility of extending the shelf lives of food products by reducing microbial populations, and has been evaluated as a method for modifying the techno-functional properties of plant proteins [67], including dry fractionated lentil protein concentrate [86] and yellow lentil protein concentrate [87]. The application of HPP can alter the gelling and swelling ability, solubility, viscosity, and foam/emulsion stability of legume proteins by modifying their structure via pressure-induced denaturation [88]. Therefore, considering that proteins are known to bind flavor compounds by trapping them physically or via chemical interactions [67], some research studies have evaluated the effect of HPP on flavor. The ability of HPP to reduce the interactions between volatile compounds due to structural modifications has been observed in plant proteins by Bi et al. [89] and Houde et al. [90]. Moreover, HPP is able to inactivate LOX [91].

The effect of different values of pressure has been studied in pea milk, pointing out that treatments at 550 MPa led to retaining a greater amount of hexanal than those at 200 MPa [89]. However, no study to date has specifically evaluated the effect of HPP on the volatile profiles of lentil proteins or lentil-based foods, despite the fact that this technology has much potential to be explored as a de-flavoring strategy.

### 5.3. Variation in pH

The principal method for obtaining legume protein isolates is wet extraction, based on the dispersion of flour in water at pH 9 to solubilize proteins, followed by isoelectric precipitation at pH 4.5–4.8 [92]. Similar to other enzymes, LOX has a different pH optimum based on different isoforms [93]. The wet extraction of protein from legume flour can, therefore, impact the LOX activity and, consequently, influence some key components of the beany flavor. In addition, changing the pH or adding salt during protein extraction can affect the protein structure, hydrophobicity, and surface charge, altering flavor–protein bonds and promoting decreased off-flavors [28].

Few studies have investigated the influence of pH on the volatile profile of legumes, being primarily focused on soy and peas. Gao et al. [94] compared pea proteins obtained by alkaline extraction at pHs of 8.5, 9.0, and 9.5. They found that the activity of LOX was reduced with an increase in the pH, lowering the concentrations of the volatile compounds typically associated with the beany flavor. Similarly, Iassonova et al. [95] compared different pH treatments and observed the highest levels of hexanal, 1-octen-3-ol, and nonenal in soybeans at pH 6.8 and lower concentrations of these volatiles at higher or lower pH values.

In view of the growing interest in protein foods enriched with legume isolates and concentrates, obtained by wet methods, the influence of pH also needs to be studied in depth in lentil proteins, as it may have an impact on the overall characteristics of the final product. Interestingly, pH shifting has been investigated as a strategy to modulate the quality of meat analogues [96], highlighting the needs of examining, in depth, the possible influence on the formation of volatile compounds.

### 5.4. Alcohol Washing

The washing of legume flours or protein concentrates using alcoholic solutions (ethanol at 20, 50, and 80% *v*/*v*, with water) improves their flavor profiles due to the alcohol solubility of several volatile compounds (e.g., hexanal, nonanal, heptanal, 1-penten-3-ol, 2-penten1-ol, and butanone), reducing beany and green off-flavors. Ethanol also alters the enzymatic activity of LOX. The best ethanol concentration for this purpose was found to be 80% [68]. Additionally, the washing process removes impurities, including simple sugars, oligosaccharides, fats, and ash, contributing to achieving cleaner and more refined legume flours.

Tan et al. [97] suggested that washing with ethanol reduced the 1-hexanol in pea proteins when using appropriate concentrations. However, at 15% and 25% *v*/*v*, the LOX activity was not reduced, with increases in hexanal, 1-octanol, 1-nonanol, 1-octen-3-ol, trans-2-hexenal, and nonanal, which are associated with beany, green, and earthy off-flavors. Instead, lentil protein isolate washed with ethanol 75% (*v*/*v*) at a ratio of 1:10, *w*/*v*, then centrifuged and vacuum-dried, was effectively stripped of off-flavors [98]. An influence of vacuum-drying could not be excluded, with this technique being known to allow for de-flavoring [82]. Isopropanol was also proposed, but it is not a viable option, as it is not food grade.

Ethanol extraction can also be combined with the supercritical CO_2_ extraction of flavor compounds, which was used to de-flavor pea flour. This process must be studied to identify the optimum conditions (in this case, 22% ethanol, 86 °C, and 42.71 MPa) in order to maximize the de-flavoring effect [99]. Also, the particle size of flour has to be considered [100], because it can influence the diffusion of the solvent within particles and, consequently, the extraction rate.

These results show that washing with alcohol can be effective, but highlight that preliminary tests are needed to define the optimal alcohol concentration, even when used in combination with other extraction systems. Moreover, the physical-chemical properties may change after alcohol washing, with a reduction in protein solubility often being observed [25,35]; therefore, the suitability of this treatment must be verified case by case, also considering the economic and environmental sustainability of the process.

### 5.5. Addition of Adsorbents

The use of solid adsorbents and membrane filtration is a promising nonthermal physical treatment that can de-flavor lentil ingredients. This process consists of the use of adsorbent material, generally resins, to de-flavor the product. This approach was tested on lentil protein isolates by Guldiken et al. [69], who used synthetic resins (Amberlite XAD16N, Amberlite XAD7HP, Amberlite XAD4, Sepabeads SP207, and Diaion HP20) constituted by polystyrene/divinylbenzene or acrylic ester, with varying pore sizes ranging from 50 to 580 Å. The lentil protein isolate was solubilized in water at 5%, adjusting the pH at 9.5, then the resins were added, keeping a 1:0.5 *w*/*w* ratio between the protein and the adsorbent. This was followed a filtration step, pH correction, and lyophilization. Differences in the effectiveness of de-flavoring were observed, depending highly on the properties of the absorbent material, such as its polarity, surface area, matrix, and pore diameter. In particular, Amberlite XAD16N, Sepabeads SP207, and Diaion HP20 reduced the major aldehydes by 32–41% and, specifically, hexanal by 39–52%, in the following ascending order of effectiveness: Sepabeads-SP207 < Amberlite-XAD16N < Diaion-HP20. 3,5-Octadien-2-one was markedly reduced by all adsorbents (by 61–92%), as well as alcohols (by 80–84%) and 2-pentylfuran (by about 50%).

The addition of β-cyclodextrin is another possible way to reduce the flavor of lentil ingredients, as this molecule is able to remove volatile compounds by trapping them in its inner core (complex formation). Treatment with β-cyclodextrin is similar to the treatment with adsorbents. It requires first solubilizing the lentil ingredient, then adding the β-cyclodextrin. Finally, the suspension must be filtered to remove the complexes, and the protein must be dried. The de-flavoring effectiveness of β-cyclodextrin has been demonstrated in soybean and pea proteins [50,101], but has not yet been tested in lentil.

Further investigations should also be carried out to explore the potential application of adsorbent-based methodologies on a large scale, by integrating an adsorption step in the regular productive process of protein isolates by wet extraction. In addition, water and energy consumption could be a limitation, since dry fractionation has been recently suggested as a more sustainable procedure compared to wet extraction for obtaining pulse protein concentrates [102,103].

### 5.6. Germination

Germination is the process in which a dormant seed begins to sprout, and a radicle (the embryonic root) and shoot emerge and start to grow into a young plant. This economical and traditional process involves benefits associated with a reduction in antinutrients and increases in the B vitamins and antioxidant activity of lentils [6,73]. Moreover, germinated lentil flours have an increased water absorption and foaming activity compared to regular lentil flours [70,71,104]. Compared with other de-flavoring techniques, which, to date, have generally not been extensively studied on lentils, numerous studies have instead evaluated the effect of germination on this legume, driven by the nutritional interest of this technique. Some of these have also examined the evolution of flavor.

During germination, the structure of the endosperm is disrupted with germination, which could allow for an easier removal of protein-bound flavors. However, the activation of enzymes, including LOX, may induce biochemical changes such as lipid and protein degradation and oxidation [72]. The overall volatile profiles of lentils can be complexified with germination, so this process cannot always be properly termed a de-flavoring technique. Moreover, conflicting results are sometimes reported among studies (see Table 3), resulting either in a desirable or non-desirable change in flavor, probably attributable to the different lentil varieties considered (with different chemical compositions), the use of dehulled or non-dehulled lentils, and different conditions for both germination and post-germination treatments. However, it appears that, by controlling the germination conditions, particularly the germination time (keeping it short) and post-germination treatment (preferring freeze-drying over oven-drying at a medium temperature), it is possible to achieve a decrease in beany flavor [72].

No post-germination treatment was conducted in a study focusing on assessing the sensory properties of fresh sprouts prepared by germinating (20 °C, 99% RH, in the dark) green lentils for seven days, with sensory checks performed every 24 h [7]. This study highlights the pure effects of germination without the influence of subsequent treatments. The panelists assessed a significant decrease in beany flavor after five days of germination. Further prolonging the germination for six and seven days did not induce further significant reductions. However, other undesirable flavors behaved differently. In detail, the grassy flavor increased after three days, then remained constant; the green flavor did not change over the entire period of germination; and off-flavors increased from the sixth day of germination onward. Although this study did not evaluate volatile compounds, so could not give information from the chemical point of view, it is undoubtably valuable due to the importance of sensory features for any food product. Indeed, it must be emphasized that germination may lead to inherent beany flavor decreases, but some new flavors that could also be undesirable could increase. Overall, it seems that keeping germination times below three days prevents the occurrence of other unpleasant flavors.

The negative effect of a prolonged germination time was also confirmed by Rajhi et al. [43], who found that germinated lentil flours (at 20 °C and 99% RH in the dark for five days, followed by drying at 45 °C for 6 h and grinding) showed an increase in 2-pentyl furan, nonanal, and decanal compared with raw seeds. Interestingly, pentyl furan appeared after the grinding of germinated seeds due to exposure to oxygen during this process, highlighting that all necessary steps after germination should be carefully considered (grinding and drying), as they have an influence on volatiles.

Flours prepared from green lentils that were germinated for two days (22–24 °C under a wet cloth, in the dark, after overnight soaking, followed by oven drying at 60 °C for 17–18 h) [70,71] showed a decrease in beany flavor after both 1 and 2 days of germination, but with better results after 1 day [71]. An increase in aldehydes compared to non-germinated flour (with a greater increase after 24 h and a lower increase after 48 h), as well as an increase in alcohols (more elevated after 48 h than 24 h), was ascertained. In detail, strong decreases in nonanal and 2-pentyl furan (both without significant differences between 24 and 48 h) and slight increases in the concentrations of hexanal (more present after 24 h than after 48) and heptanal were observed (more abundant after 48 h than 24 h) compared to the non-germinated flour. Among alcohols, 1-hexanol and 1-octen-3-ol increased, with the latter being not detected in non-germinated flour and progressively increasing with a longer germination time.

In addition to flour, lentil protein isolate is another semi-processed ingredient widely marketed today. The olfactometric profiles of protein isolates extracted from lentil seeds germinated for only one day (25 °C, 99% RH in the dark, followed by freeze drying and grinding to flour), compared with protein isolate obtained from non-germinated seeds, showed reductions in some volatile compounds associated with a beany flavor, while others very slightly increased. All the beany volatiles saw marked increases in their concentrations with longer germination times (3–5 days) [72]. In detail, nonanal, 3-octen-2-one, heptanal, and (*E*, *Z*)-2,6-nonadienal were reduced after 1 day of germination and, with the exception of nonanal, which remained at a low concentration, all increased with longer germination times. Hexanal and (*E*)-2-octen-1-ol increased very slightly with one day of germination, but their level increased much more at longer germination times [72]. Therefore, since the beany flavor of protein isolates improved with short germination (24 h) but intensified significantly if germination continued, care must be taken in timing.

Germinated lentils have been also proposed for preparing a plant-based beverage. In this case, no significant differences were found in the legume odor and flavor and in the overall sensory quality of the beverage compared to a control prepared with non-germinated lentils. In detail, the nonanal content decreased and (*E*, *E*)-2,4-nonadienal content increased, while hexanal and pentanal remained unchanged with the germination (25 °C for three days) and preparation of the beverage (sterilizing at 121 °C for 15 min, mixing with water at a ratio of 1:9, blending, and filtering) [73]. The addition of raspberry–cranberry pulp was proposed as an easy and effective way to improve the sensory features of this beverage.

A recently published study outlined the possibility of extrusion cooking germinated lentils to prepare meat analogs [65], achieving a reduction in volatile compounds. Germination and extrusion had a synergistic effect, reducing some volatile compounds responsible for the beany flavor, such as hexanal, 1-hexanol, 2-nonenal, (*E*)-2-octen-1-ol, and (*E,E*)-2,4-nonadienal. In parallel, other volatile compounds could be formed under these conditions, such as the products of the Maillard reaction, typically characterized by pleasant nutty and roasted flavors.

### 5.7. Fermentation

Fermentation is the oldest biotechnological approach applied to food and beverages, involving the use of spontaneous or selected microorganisms, typically bacteria and/or yeasts, which metabolize carbohydrates and other compounds present in the matrix. Fermentation enhances nutrient bioavailability and protein digestibility, reduces antinutrients, such as phytic acid and protease inhibitors, promotes antioxidant activity, and reduces carbohydrate content. Probiotic lentil-based fermented beverages prepared using *Lactobacillus* strains were proposed by Verni et al. [17]. Boeck et al. [105] fermented a yogurt alternative using lentil protein isolate and selected strains of lactic acid bacteria. A sensory evaluation of the final product scored the “beany flavor” from 4.4 to 6.1, depending on the strain, on a 0–10 scale. Canonico et al. [106] used wild yeasts to produce a functional craft beer with reduced alcohol content, fortified with hydrolyzed red lentils as a protein source. They found that specific yeast strains resulted in increased aroma compounds compared to traditional *Lactobacillus thermotolerans*/*S. cerevisiae* cultures, influencing the sensory profile by imparting a fruity aroma. Perri et al. [107] carried out the fermentation of whole and sprouted lentils using selected lactic acid bacteria (LAB), resulting in sourdoughs that were later incorporated into bread formulations. The inclusion of sourdough had a significant effect on the volatile profile of the bread, improving its flavor.

Fermentation can impart unique flavors and aromas. Through their enzymes, microorganisms degrade proteins, lipids, and complex carbohydrates, releasing their basic components (peptides, amino acids, fatty acids, and simple sugars), which can be precursors to flavors and aroma compounds as fermentation proceeds, modifying the volatile profiles of the starting lentils [74]. The effect on the volatile profile is strongly related to the microbial composition, the compositional characteristics of the lentils used, and the fermentation parameters. Therefore, optimization is always needed to ensure that the variations in volatiles induced by fermentation mitigate the beany and grassy notes associated with hexanal and other products of lipid and protein degradation, contributing to the overall sensory quality of fermented lentil products. Fermentation is relatively inexpensive and requires minimal or no chemicals, but can be time-consuming.

Solid-state fungal fermentation with *Pleurotus ostreatus* (28 °C for 14 days) improved the volatile profile of lentil flour of the cultivar Pardina [77] by markedly reducing 1-hexanol and hexanal compared with non-fermented lentils, while benzaldehyde and 3-octanone (absent in the starting lentils) strongly increased. These compounds varied with successive oven-drying or freeze-drying, with 3-octanone decreasing and benzaldehyde (giving a pleasant almond flavor) further increasing, while hexanal and 1-hexanol remained unchanged. In lentils of the Castellana cultivar, instead, hexanal increased with fermentation, but then decreased with drying, while benzaldehyde strongly increased but then decreased with drying. Nonanal, not detected in Pardina, increased with the fermentation of Castellana lentils but decreased with successive drying. The volatiles were, therefore, significantly influenced by both the lentil cultivar and the post-fermentation operations.

Several volatiles, such as hexanal, 1-hexanol, hexanoic acid, 2-pentyl furan, and (*E*,*E*)-2,4-nonadienal, monitored during the solid-state fermentation of green and red lentils, were found to be significantly correlated with microbial counts and pH [47]. Lentils subjected to solid-state fermentation with *Pediococcus acidilactici* showed a more complex volatile compound profile than lentils subjected to submerged fermentation with the same strain [108]. Compared to non-fermented lentils, hexanal was markedly reduced by submerged fermentation, while it remained almost unchanged with solid-state fermentation. Nonanal did not change with either fermentation type. Benzaldehyde increased more with solid-state than with submerged fermentation.

Kryachko et al. [109] focused on microorganisms able to produce cyclodextrins (described in Section 5.5). The authors proposed a two-stage process starting with acid treatment to allow for volatile compounds to be released by legume proteins, followed by washing and redissolving the protein precipitate, fermentation with cyclodextrin-producing microorganism(s), and the removal of the precipitated cyclodextrin complexes with flavor compounds. Alternatively, enzymes such as cyclodextrin glycosyl transferases (CGTases) can be used to remove off-flavors [110]. These treatments have not been applied to lentil ingredients; therefore, studies should be conducted with this purpose.

### 5.8. Breeding for Low LOX and Lipids

Possible strategies to mitigate the beany flavor of lentil and lentil-based products could involve the genetic selection and utilization of varieties with low contents of the volatile compound precursors responsible for off-flavors and/or low enzymatic activity. In fact, the lipid content and fatty acid composition are significantly influenced by the variety [111]. Furthermore, lentil is known to have high LOX activity, which is quite variable among different cultivars [112]. It has also been observed that tannin extract from the lentil seed coat counteracts the lipid peroxidation catalyzed by lipoxygenase and β-carotene, suggesting an increase in the phenolic compounds in lentil [113]. However, the antinutritional effect of tannins is a major drawback.

Genome editing with Clustered Regularly Interspaced Short Palindromic Repeats-associated protein 9 (CRISPR-Cas9) technology has been recently applied to edit LOX-related genes in soybean [114] and pea [115]. The developed soybean lines were LOX-free and still await further evaluation. The pea lines showed a significant reduction in LOX activity and, in turn, in the concentrations of key volatiles, such as hexanal, 2-hexenal, heptanal, (*E*)-2-heptenal, 1-octen-3-ol, octanal, (*E*)-2-octenal (*E*,*E*)-2,4-nonadienal, and 2-pentyl furan. Modulating enzyme activity may be a better strategy, since LOX plays a role in plant physiology; hence, its absence may affect agronomic characteristics.

No studies aimed at lowering the LOX activity of lentil or its lipid content have been conducted so far. Future research activities could more thoroughly study the effect of lentil composition on the development of volatile compounds in food and, consequently, set up specific molecular breeding programs for lentils for improving their traits, such as beany flavors.

## 6. Improvement of Communication Strategies

Lentils are popular in Asia, the Middle East, and in Mediterranean countries, but are much less commonly consumed in Western countries, especially the US and Canada [116,117]. Barriers to the consumption of lentils in Canada have been attributed, in most cases, to a lack of knowledge on recipes to make them palatable, ultimately resulting in cultural non-acceptance [117]. It should be noted that “beany” is considered as an “atypical” and unusual flavor note by consumers unfamiliar with lentils, but is less disliked when people are more familiar with pulses. In addition, millennials, more sensitive to environmental issues and climate change, may be more open to appreciate legume-based foods that are sustainable compared to those of animal origins. Lentils snacks were found to be appreciated by Spanish millennials, with a liking score of 7.0 over a 9-point hedonic scale [118].

Although plant-based foods (including lentil-based foods) specifically designed to mimic their conventional counterparts (milk and meat analogs) are an emerging trend, the sensory perception of these products remains a major challenge to be addressed. In fact, while consumers may expect these products to lack the “foreign” sensory notes associated with legumes and to perfectly mimic other sensory characteristics, achieving this perfect imitation often requires the addition of various additives and ingredients, potentially reducing acceptance by consumers seeking “clean label” foods. However, new horizons for the food industry are opening up thanks to the application of modern tools such as artificial intelligence, which is useful for formulating original food products suited to individual tastes and discovering the best flavor combinations while optimizing consumer acceptance and taking into account nutritional goals and economic aspects [119].

In addition, as we are experiencing the rapid development of these product categories, perhaps the time is ripe for plant-based foods to be communicated for their identity and sensory characteristics, without presenting them only as an imitation of other foods. The promotion of nutritional education, incentive strategies such as free tasting, and appropriate communication when launching new food products containing legumes, highlighting their nutritional benefits [120], could positively influence consumer acceptability and, at the same time, make their sensory properties more familiar. Therefore, it can be hypothesized that future consumer trends may focus more on the specific identity and sensory characteristics of lentil-based foods, broadening consumer awareness, promoting consumption, and depowering the importance of de-flavoring.

## 7. Conclusions and Future Perspectives

Lentil-based products are already being marketed and consumed, and food companies are investing in the development of new fortified formulations for market launch. However, the sensory impact of such foods should be carefully evaluated and modulated, as it is responsible for the success of these products.

This review provided a detailed analysis of the volatile compounds of lentil-based ingredients and strategies for mitigating their associated off-flavors. Assuming that the appropriate storage and processing are conducted, so as to prevent or limit undesired oxidative phenomena, several treatments are available: thermal, non-thermal, and biotechnological, all able to reduce the beany flavor. However, the literature review showed that lentil is less studied than other legumes, so more research should be conducted, because varietal differences in responses to various de-flavoring treatments occur, so it is not possible to directly translate the results obtained with other legumes to lentil. Indeed, innovative technologies with great potential, such as high-pressure processing (HPP) and the use of adsorbents, have not been explored in detail or are still totally unexplored in lentil.

Further research should also be carried out to better understand the economic implications of these various de-flavoring treatments. Conducting comprehensive assessments on the costs and benefits of different techniques is essential, considering their impacts on final pricing and industrial scalability.

In parallel, to prevent the formation of off-flavors, instead of eliminating them, the development of lentil varieties with a low LOX and lipid content, as is currently underway for soybean and pea, would significantly reduce the volatile compounds forming from lipid oxidation.

Finally, as the field of innovative lentil-based foods is growing rapidly, a question arises: will increased consumer awareness of the nutritional benefits and greater knowledge of the inherent sensory characteristics of lentil-based foods reduce the importance of de-flavoring in the future?

## Figures and Tables

**Table 1:** Principal undesirable flavor/aroma notes, descriptions, and associated volatile compounds identified in lentil flours, protein isolates, concentrates, and derived foods.

Undesirable Flavor/Aroma Note *	Description	Volatile Compounds	Origin	References
Beany	Pea-like, musty/earthy, musty/dusty, sour, nutty, brown	(*E*,*E*)-2,4-heptadienal, 1-octen-3-ol, 1-octen-3-one, 3-octen-2-one, 2-pentyl furan, 3-methyl-1-butanol, 2-butanone, 2-pentanone, 2-hexanone, 3-isobutyl-2-methoxypyrazine, and 2-isopropyl-3-methoxypyrazine	Inherent in legume seeds, protein and amino acid degradation, oxidation of fatty acids during storage and processing	[26,28,43,44]
Grassy	Cut grass off-flavor, greasy and fatty	Hexanal, heptanal, nonanal, (*Z*)-3-hexen-1-ol	Inherent in legume seeds, oxidation of fatty acids during storage and processing	[26,43]
Green	Fresh green pea	Hexanal, heptanal, nonanal, 2-pentyl furan	Inherent in legume seeds, oxidation of fatty acids during storage and processing	[26,43]
Rancid	Aged oil and fat	Hexanal, heptanal, octanal, (*E*)-2-heptenal (*E*)-2-octenal, nonanal, (*E*)-2-nonenal, (*E*,*E*)-2,4-nonadienal, (*E*)-2-hexenal, (*E*,*E*)-2,4-decadienal, and pentanal **	Oxidation of fatty acids during storage and processing	[35]
Brothy	Boiled beef, cooked potatoes, cooked vegetables	Methional	Degradation of methionine during processing	[26]
Sulfur	Metallic, cabbage, egg, onion	Dimethyl sulfide and dimethyl trisulfide	Degradation of methionine and cysteine during processing	[26]

* According to orthonasal (aroma and odor) or retronasal (flavor) olfaction; ** Combinations of these compounds may result in a beany flavor.

**Table 2:** Treatments applicable to lentil flours and lentil-based ingredients to mitigate flavor.

Type of Treatment	Mechanism of Action	Advantages	Disadvantages	References
Heat treatment (pre-cooking, roasting, and drying)	Reduction in LOX activity; volatilization of off-flavors; new flavors	Low investment cost; operational ease and simplicity	Energy consumption; possible color alterations	[62,63]
Extrusion cooking	Volatilization of off-flavors; new flavors	Short processing time	Energy consumption; investment cost	[64,65]
High-pressure processing	Reduction in LOX activity	Short processing time	Investment cost; energy consumption; water consumption	[66,67]
Alcohol washing	Reduction in LOX activity; solubilization of off-flavors	Low investment cost; operational ease and simplicity	Chemical residues; reduction of protein solubility	[68]
Variation in pH	Reduction in LOX activity	Low investment cost; operational ease and simplicity	Chemical residues	[28]
Adsorbents	Physical removal of off-flavors	Low investment cost; operational ease and simplicity	Chemical residues, energy consumption; water consumption	[69]
Germination	New flavors	Low investment cost; operational ease and simplicity; minimal or no chemicals needed	Possible oxidations	[7,43,70,71,72,73]
Fermentation	New flavors	Low investment cost; operational ease and simplicity; minimal or no chemicals needed	Long processing time	[74]

**Table 3:** Effect of germination on the volatiles of lentil flours, protein isolates, and lentil-based beverage.

Lentil Product	Type of Lentil	Germination Days	Germination Conditions	Post-Germination Treatments	Beany Volatile Compounds Involved		Effect on Beany Flavor	Reference
					Increasing	Decreasing		
Lentil sprouts	Green lentil, Aldona cultivar	7	Soaked 3.5 h at RT, then kept at 20 °C, 99% RH, in the dark	None	n.d.	n.d.	Decreasing	[7]
Lentil flour	Brown and green lentil	5	Dehulled, then sown in Petri dishes at 20 °C, 99% RH, in the dark	Drying at 45 °C for 6 h and grinding	2-Pentyl furan, nonanal, and decanal	-	Increasing	[43]
Lentil flour	Altamura lentil, not dehulled	1	Soaked overnight, then kept at 22–24 °C, under a wet cloth, in the dark	Drying at 60 °C for 17–18 h and grinding	Aldehydes and alcohols	2-Pentylfuran	Decreasing	[70]
Lentil flour	Altamura lentils, not dehulled	2	Soaked overnight, then kept at 22–24 °C, under a wet cloth, in the dark	Drying at 60 °C for 17–18 h and grinding	Aldehydes and alcohols	2-Pentylfuran	Decreasing	[70]
Lentil flour	Green Altamura lentil	1	Soaked overnight, then kept at 22–24 °C, under a wet cloth, in the dark	Drying at 60 °C for 17–18 h and grinding	Hexanal, heptanal, and 1-octen-3-ol	2-Pentylfuran and nonanal	Decreasing	[71]
Lentil flour	Green Altamura lentil	2	Soaked overnight, then kept at 22–24 °C, under a wet cloth, in the dark	Drying at 60 °C for 17–18 h and grinding	Hexanal, heptanal, 1-octen-3-ol, and 1-hexanol	2-Pentylfuran and nonanal	Decreasing	[71]
Protein isolate	Commercial, not specified	1	Soaked 5.5 h, then kept at 25 °C, 99% RH, in the dark	Freeze-drying and grinding, then wet extracting the protein	Hexanal and (*E*)-2-octen-1-ol	Nonanal, 3-octen-2-one, heptanal, and (*E*, *Z*)-2,6-nonadienal	Decreasing	[72]
Protein isolate	Commercial, not specified	3–5	Soaked 5.5 h, then kept at 25 °C, 99% RH, in the dark	Freeze-drying and grinding, then wet extracting the protein	Hexanal, (*E*)-2-octen-1-ol, nonanal, 3-octen-2-one, heptanal, and (*E*, *Z*)-2,6-nonadienal	-	Increasing	[72]
Lentil-based beverage	Brown lentils	3	At 25 °C in a commercial sprouter	Sterilizing at 121 °C for 15 min, mixing with water at a ratio of 1:9, blending, and filtering	(*E*, *E*)-2,4-nonadienal	Nonanal	Unchanged	[73]

RH = relative humidity; RT = room temperature; and n.d. = not determined.

## Data Availability

No new data were created or analyzed in this study. Data sharing is not applicable to this article.
